# Case of Cholera Combined with Symptoms of Remittent Fever

**Published:** 1832

**Authors:** James C. Finley


### Art. III.—*A case of Cholera, combined with symptoms of Remittent Fever.* By James C. Finley; M. D.

On Monday, the 14th of October I was requested by L. S., a button-maker, to prescribe for a diarrhœa, under which he had been labouring for some days. I gave him two pills of opium and calomel, one grain of the former to five of the latter, with directions to take one immediately, the other in the afternoon, and in the evening, to take a dose of the compound syrup of rhubarb.

On the 16th I was sent for early in the morning, and found that the syrup of rhubarb, taken on the afternoon before, had operated actively, producing copious serous evacuations. He was now very sick at the stomach, vomiting frequently, and dissharging from the bowels often, though not very copiously, the *rice-water* discharges. The face was sunken not livid, but of a smoky complexion, easily associated in the mind with the appearance of the sun in Indian summer. An emetic, of three tea-spoonsful of the flour of mustard, in a half pint of warm water, was immediately administered, which, after emptying the stomach, relieved the nausea and diminished the catharsis,a large sinapism was applied over the abdomen, and warm bricks and frictions assiduously applied to the extremities;and brandy toddy, for which he had a strong instinctive appetite, was administered in as large doses as his stomach would bear, and a pill, of calomel 5 grs. opium 1 gr. was given every hour until reaction took place.

These means, after a lapse of several hours, being followed by no alleviation in the symptoms, a vein was opened in the arm. The blood flowed from the arm of the usual color of venous blood, but after a few ounces were lost, became of a scarlet color. After the loss of about 6 ounces, the pulse sunk, and the vein was stopped. After the bleeding he was evidently worse; the pulse weaker, and the restlessness increased. Cupping was now resorted to. Several tumblers were placed over his chest and abdomen, and renewed at short intervals. At about 2 o'clock, P. M, the system began to react, the pulse became fuller, the skin warmer, and he passed, comparatively, a comfortable night. About 5 o'clock on the next morning, however, the symptoms of depression again returned, the rice-water discharges, which had only occurrred at long intervals during the preceding afternoon and night, became more frequent, the countenance became more ghastly, and the symptoms, in every respect, more unfavourable than on the day before

At noon Dr. Staughton saw him with me, and recommended a continuance of the same treatment. Reaction again took place about the same time, and the symptoms during the afternoon and evening were not materially different from those of the preceding day. On the following morning, however, at about the same hour, the symptoms of depression again returned. At the suggestion of Dr. Staughton, 15 grains of quinine were taken in the course of the morning, in three doses. Doubtful of the necessity or propriety of using such a quantity of brandy, good Madeira wine was substituted for it, of which, in the course of six hours, he drank nearly a quart. It did not, however, appear to supply the necessary stimulus, and we supposed it necessary to again recur to the brandy. The depression on this day was more alarming than on the preceding days, but when reaction took place it assumed a more satisfactory and decided character. Great pains were taken on the following day to guard against the periodical depression, but it did not recur. The restlessness and the jactitation, as well as the desire for the brandy, continued during this and the following day. We now, for the brandy, by degrees substituted porter, for which he had a strong desire, and accommodating his diet to his increasing strength, he soon regained his health, and returned to his ordinary business.

On the afternoon of the third day we derived great benefit, from the administration of pills of assafœtida, which were given, by the advice of Dr. Staughton. The uncomfortable distension, arising from flatulence, and the restlessness and jactitation, were more relieved by it, than by any other remedy that he had taken in the course of his disease.

Mr. S. is a man of strictly temperate habits, and therefore the great quantity of brandy which he consumed was the more remarkable. He called for it incessantly, both in the stage of depression and reaction, and the quantity consumed was not less than a quart in twenty-four hours, for four days. Whether this was a healthy or a morbid appetite, I pretend not to decide. I did not, however, consider it safe to neglect the indication of treatment which it held out.

After the second day he also expressed a strong desire for porter. It was at length given to him, and with a good effect. Since that time I have met with another case, in a confirmed toper, who revolted at the bare mention of brandy, but who eagerly called for porter. As soon as good London porter could be procured it was given to him, in quantities suited to the irritability of his stomach, and with a most admirable effect. The retching and jactitation, which were very distressing, were relieved almost immediately.

In the case which we are describing, the bile began to appear in the discharges on the afternoon of the second day, and continued to increase until convalescence was established. He had had, before this, one period of reaction lasting more than 12 hours; and afterwards, a stage of depression followed, more severe than any that had gone before. The prudent physician will not, therefore, be disposed to relax his exertions on account of the appearance of this symptom of convalescence. In one of our patients a relapse took place, and the patient sunk under a dark bilious diarrhœa; while in another, on the contrary, there was no appearance of bile in the discharges, until four days after convalescence was decidedly established.

